# Diagnostic algorithm for determining primary tumor sites using peritoneal fluid

**DOI:** 10.1371/journal.pone.0199715

**Published:** 2018-07-19

**Authors:** Cheol Keun Park, Douglas P. Malinowski, Nam Hoon Cho

**Affiliations:** 1 Department of Pathology, Severance hospital, Yonsei University College of Medicine, Seoul, Republic of Korea; 2 Department of Pathology, Armed Forces Capital Hospital, Seongnam, Republic of Korea; 3 Women’s Health and Cancer, BD Life Sciences, Durham, North Carolina, United States of America; University of South Alabama Mitchell Cancer Institute, UNITED STATES

## Abstract

This study was conducted to develop a novel algorithm for determining the origin of tumors by combining analysis of cluster patterns with immunocytochemistry (ICC) for markers in cells from fine-needle aspirates of ascites. We used LBC, based on SurePath^TM^ (BD Diagnostics) technology, to screen 96 peritoneal fluid samples from patients with known malignancies and from 10 control patients with cirrhosis. Following dual ICC staining for cytokeratin 7 (CK7) and paired box gene 8 (PAX8), we developed an algorithm using immunoreactivity and three-dimensional (3D) cluster patterns to correlate staining and 3D cluster patterns with common primary origins that included stomach, ovarian, pancreatobiliary tract, colon, lung, and breast cancers. With the application of an automatic digitalized image analyzer, competence performance was analyzed using receiver operating characteristics (ROC) curve analysis. CK7 and PAX8 staining and 3D cluster patterns were used to differentiate primary origins. Samples from patients with stomach cancer were no 3D cluster /CK7^+^/PAX8^-^ with area under the curve (AUC) of 0.8699 in ROC curve analysis. Samples from ovarian cancer patients were large 3D cluster/CK7^+^/PAX8^+^ with AUC of 0.9812. Samples from pancreatobiliary tract cancer patients were small 3D cluster/CK7^+^/PAX8^-^ with AUC of 0.8772. The remaining cancer samples, including breast, lung and colon cancer samples, had similar patterns of large 3D clusters/CK7^+^/PAX8^-^ with AUC of 0.882, especially for lung cancer. SurePath^TM^ technology, using 3D cluster patterns and dual ICC for CK7 and PAX8 in peritoneal fluid samples, can provide important information for determining specific primary origins in cases of unknown primary carcinoma.

## Introduction

Ascites, excess peritoneal fluid, is common in various diseases, including serous fluids in liver cirrhosis and turbid exudative fluids in various inflammation settings, including malignant tumors in visceral and pelvic organs. The primary origin of malignant ascites can be any site beyond the peritoneum; the most common origins in our cohort were ovary, stomach, and pancreaticobiliary tract. With optimal interpretation, fluid cytology could meet clinical demands by alleviating diagnostic and therapeutic conundra.[1] However, the interpretation of fluid cytology in third spaces remains a challenging field. The spectrum is remarkably wide with regard to benign or malignant diagnoses, even between tumorous or non-tumorous diagnoses, making clear diagnoses difficult. Furthermore, determination of the type of primary tumor, even in the case of malignant conditions, may not be possible using cytology alone. Many situations encounter equivocal or obscure tumor origins based on clinical or radiological information.

With the introduction of liquid-based cytology (LBC), ancillary studies, including molecular analysis and immunocytochemistry (ICC), could be utilized to alleviate diagnostic conundra.[2–8] However, the wide application of LBC is less practical. The main reason why LBC is less easily applied resides in the practical difficulty of the involved ICC, which is relatively difficult compared to traditional immunohistochemistry. Another difficulty is the lack of well-documented biomarkers, except for those involved in HPV testing. Cytokeratin 7 (CK7) and paired box gene 8 (PAX8) are currently the best markers for determining primary origin in ovarian carcinoma, stomach cancer, and pancreatobiliary tract cancer.[9,10]

Here, we aimed to develop a novel algorithm for determining the origin of tumors by combining analysis of cluster patterns with ICC for markers in cells from fine-needle aspirates of ascites.

## Materials and methods

### Case and control sample selection

A total of 247 fluid samples were collected via aspiration from peritoneal ascites and stored in a frozen state. Within 6 months of collection, samples were screened and qualified for further evaluation. Cases with low levels of fluid, cell paucity, or ambiguous primary tumors were excluded. To confirm the primary origin of malignant peritoneal fluids, we compared cytological specimens with paired tissue specimens. When no available tissue specimens, we referred to clinical information and performed ICC for cancer-specific biomarkers.

Finally, 96 malignant peritoneal fluids (33 stomach cancer, 34 ovary cancer, 13 pancreatobiliart tract cancer, 9 lung cancer, 4 colon cancer and 3 breast cancer patients) were selected. And ten peritoneal fluid samples aspirated from cirrhosis patients were selected as a control group. The need for consent was waived and this study was approved by the Institutional Review Board of Severance Hospital (4-2016-0225).

### Preparation of unstained liquid-based cytology slides

For ICC, unstained LBC slides were prepared using the SurePath^TM^ method (BD Totalys^TM^ SlidePrep System, BD Diagnostics, Burlington, NC). Briefly, unstained LBC slides were prepared according to the manufacturer’s protocol. CytoRich Red (CRR) fixative samples were centrifuged at 3240 rpm for 5 minutes. Supernatant fluids were removed and pellets were vortexed to homogenize the sample. When no visible or small pellets were identified, representative samples (1 to 5 drops) were transferred to 5 mL buffered distilled deionized water. Following centrifugation for 5 min at 3240 rpm, supernatant fluids were decanted and pellets were vortexed for 15 seconds to homogenize samples. The samples were transferred onto the BD Totalys^TM^ SlidePrep system for processing. BD SurePath^TM^ PreCoat slides were placed on the slide racks in the same position as the tubes. Following execution of the NON-GYN program on the BD Totalys^TM^ SlidePrep system, the unstained slides were stored in 95% ethanol. This cytology slide preparation is referred to as the SurePath^TM^ method. Following cytology processing, the residual peritoneal fluid pellets were preserved at 2°C for a few months until use.

### Dual immunocytochemistry

Dual ICC was performed by using BenchMark XT Automated staining system (Ventana Medical Systems, Tucson, AZ) according to the manufacturer’s protocol. Cell Conditioning Solution (CC2; pH 6.0 citrate buffer; Ventana Medical Systems) was used for antigen retrieval. Detection was performed using ultraView Universal 3,3'-diaminobenzidine (DAB) Detection kits and ultraView Universal Alkaline Phosphatase (AP) Red Detection kits (Ventana Medical Systems). For DAB staining, primary anti-PAX8 antibody (Cell Marque, 1:30) was applied and incubated for 64 min.

Before the AP Red staining step, slides were incubated at 90°C for 4 min. For AP Red staining, anti-CK7 antibody (DAKO, 1:100) was applied and incubated for 35 min, followed by the AP Red multimer for 12 min. UltraView AP Red ISH Enhancer (Ventana Medical Systems) was applied for 4 min at 37°C. Slides were incubated for 8 min after simultaneous addition of Fast Red A and Red Naphtol (Ventana Medical Systems). Slides were incubated with Fast Red B (Ventana Medical Systems) for 8 min followed by hematoxylin and Bluing Reagent (Ventana Medical Systems) counterstain at 37°C.

### Image analysis

A Mantra Quantitative Pathology Imaging System (Perkin-Elmer, Waltham, MA) was used for acquiring multispectral images. Slides were imaged at 20× using Mantra Snap software (Perkin-Elmer). inForm™ 2.2.1 software (Perkin-Elmer) and Spotfire QPS (Tibco; Hopkinton, MA) were used for image analysis and quantification. MSI images were converted to RGB images and embedded synthetic spectrums for hematoxylin, DAB, and Fast Red were used to unmix the signals of these images. Bright field images were converted to fluorescent images for convenient image analysis. Hematoxylin spectrums were used for cell segmentation. DAB and Fast Red spectrums were used for detecting cytoplasmic areas. The optical densities of Fast Red, DAB, and cell areas from each cell were quantified. Phenotyping using machine learning was applied to divide cells into four types of cells: CK7^-^/PAX8^-^, CK7^+^/PAX8^-^, CK7^+^/PAX8^-^, and CK7^+^/PAX8^+^.

### Statistical analysis

The relationships between variables and primary origins were evaluated using chi-squared tests. To compare the performance of the selected variables, receiver operating characteristic (ROC) curves were constructed and the area under the curve (AUC) was compared. Comparisons were made using the Delong method for statistical significance of AUC.[11] Statistical analyses were performed using SAS software (version 9.2, SAS Inc., Cary, NC). *P*-values were adjusted using Bonferroni’s correction. *P* < 0.05 was considered statistically significant.

## Results

### Cytomorphological characteristics of each tumor cell cluster

**Fig 1:**
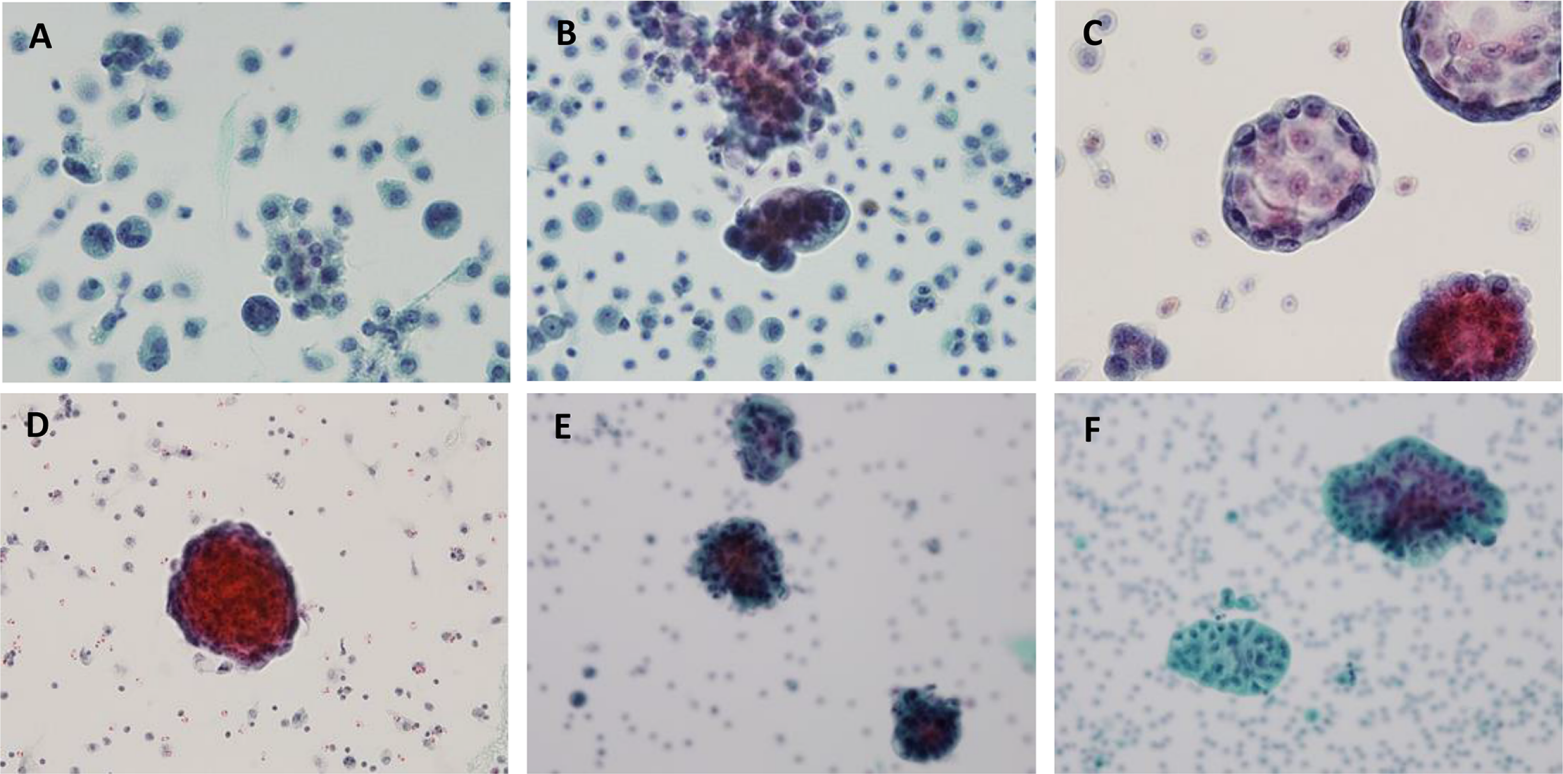
Representative cytology presentations of metastatic carcinoma. (A) stomach, (B) lung, (C) pancreatobiliary tract, (D) breast, (E) ovary and (F) colon cancer.

Cells from malignant ascites cases were prepared using CytoRich Red as a liquid-based cytology preservative. Overall, the morphology of these various malignant cells was well retained, and several key cellular features were retained in these liquid-based cytology preparations. Furthermore, obvious differences in various morpgology features could be detected among the different primary tumor cells which had metastasized to the peritoneal cavity. These features included clear morphology details of cytoplasm, nucleus, nucleolus, vacuoles and chromatin, which are summarized in Table 1. Representative images of the different tumor cell clusters are displayed in Fig 1.

**Table 1:** A summary of the key morphological features identified in metastatic carcinoma present within ascites cases.

Primary site	Cell Presentation	Key Cytology Features
Stomach	● Non-coherent cells; dispersed with no cluster	● Irregular nucleus with bilobular features. ● Foamy cytoplasm with bubbles
Lung	● Predominant, small-sized clusters and associated single cells	• Central nucleus with prominent nucleoli. • Lack of course chromatin • Mucoid vacuoles
Pancreatobiliary tract	● Small cell clusters, with uniformly round cells	• Peripheral alignment of nuclei in cell clusters • Prominent nucleoli • Fine chromatin • Mucoid vacuoles
Breast	● Large cell clusters with three-dimensional structures ● Large atypical cells ● No single cells	● Course, clumped chromatin
Ovary	● Large cell clusters; diffusely distributed ● Cells display consistent size and shape	● Prominent nucleoli ● Open chromatin ● Transparent to amphophilic cytoplasm ● Clear, protruding vacuoles ● Common borders in cell clusters
Colon	● Three-dimensional clusters of variable cell size ● Clusters are scattered	• Oval to spindle shaped nucleus • Prominent nucleoli • Lacy, ragged cytoplasm • Common border in cell clusters

### Optimization of dual immunocytochemistry

**Fig 2:**
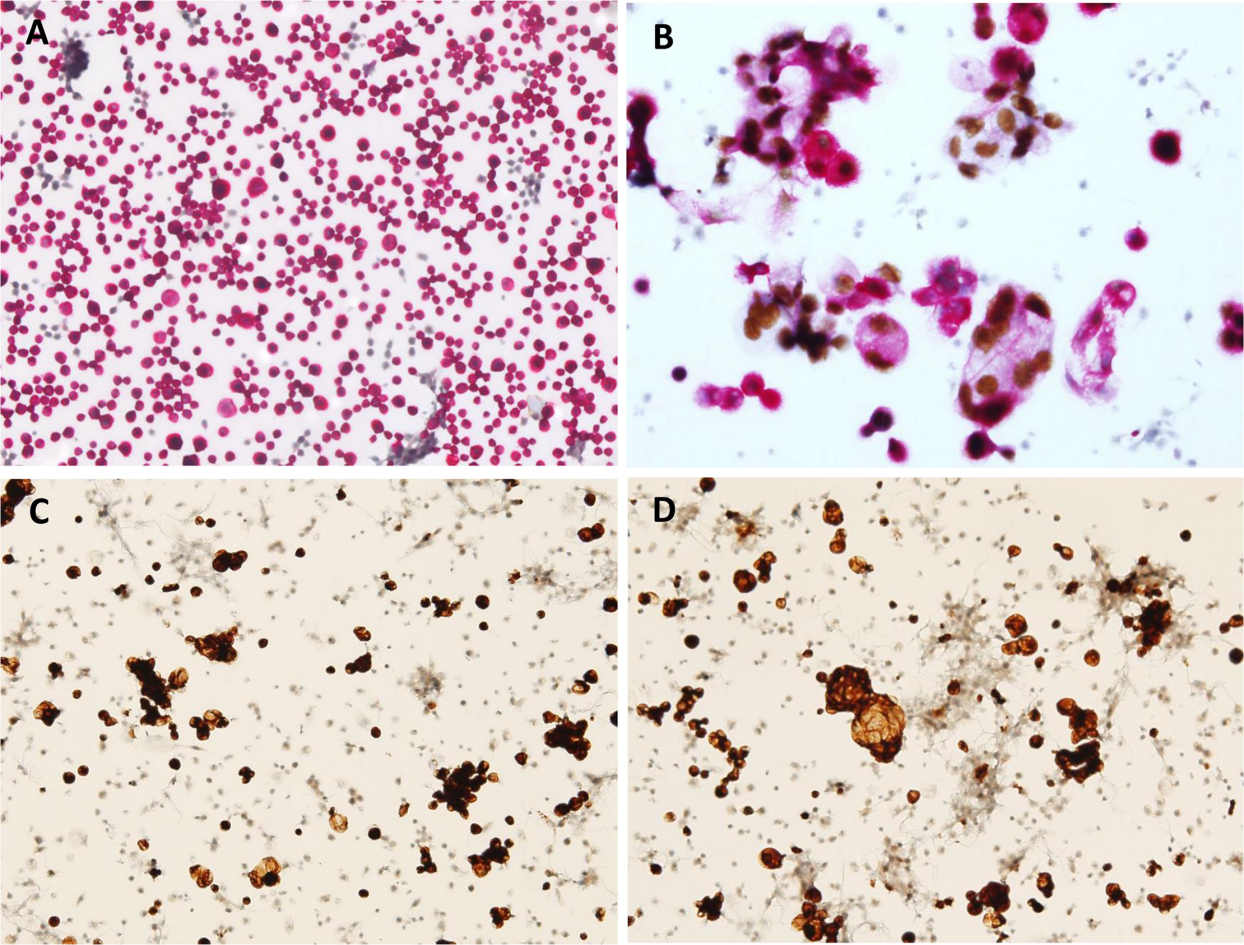
Optimizing the order of dual immunocytochemistry for CK7 and PAX8. When the antibody that is used first affects the overall manifestation because the first applied color becomes weak or even completely lost following staining with the second color. (A and B) Nuclear staining, using PAX8-specific antibody with DAB, was performed first followed by cytoplasmic or membranous staining using CK7-specific antibody with Fast Red. (C and D) CK7 staining was performed first followed by PAX8 staining. The initially applied CK7 red color becomes completely lost following DAB staining in the same case as in A and B.

In addition to morphology features, we also analyzed the pattern of cell cluster as cytomorphology in liquid-based suspensions. Cluster analysis and analysis of CK7 and PAX8 expression on cells, isolated from peritoneal fluid, were used for the prediction of primary tumor origin. We determined that performing the first nuclear staining with PAX8 ICC along with DAB, followed by cytoplasmic/membranous staining with CK7 ICC along with and Fast Red (Fig 2A and 2B), was more successful for recognizing dual signals than staining in the reverse order (Fig 2C and 2D).

### Cluster and immunocytochemistry patterns of peritoneal fluid

**Fig 3:**
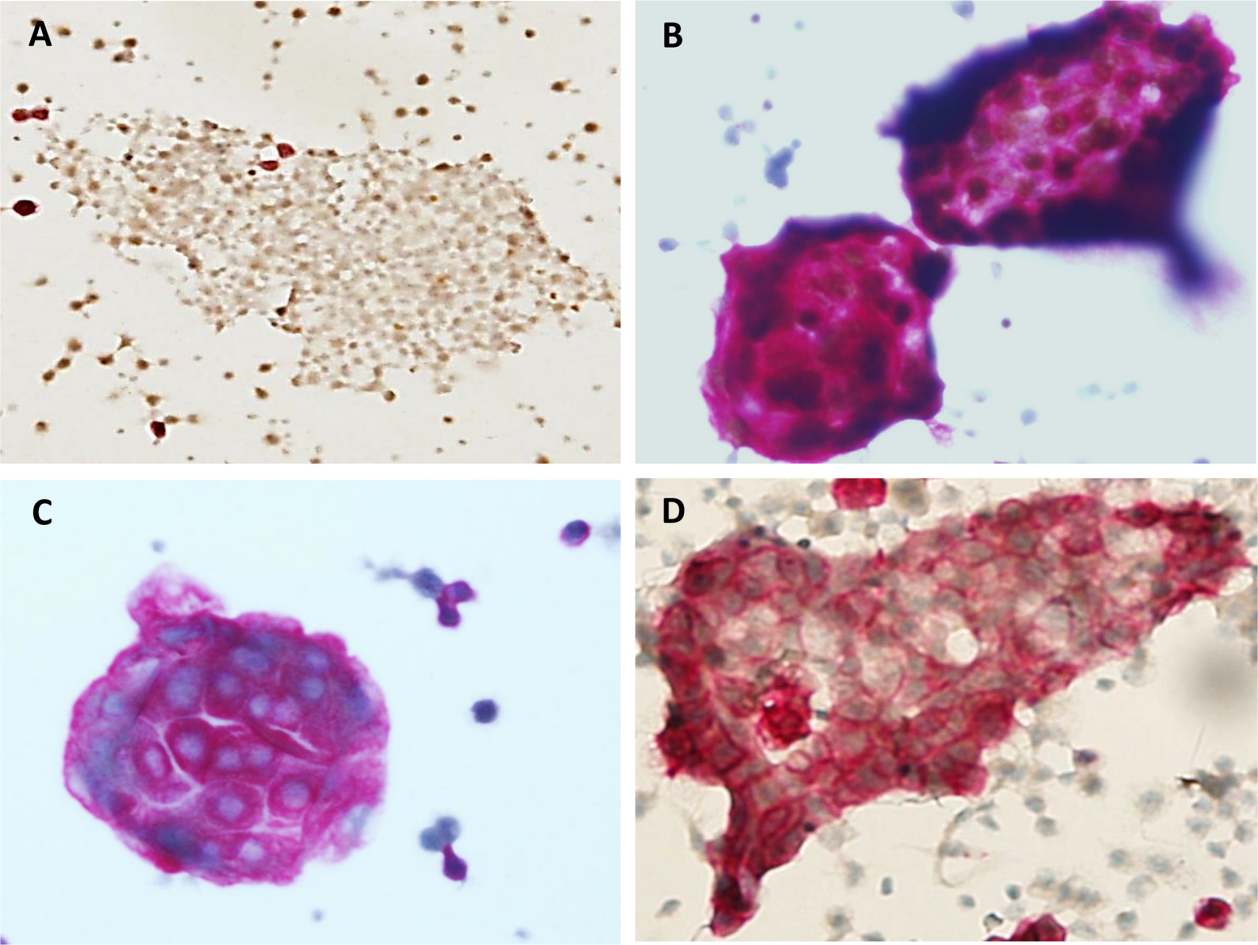
Typical immunocytochemistry patterns of cluster in peritoneal suspensions using LBC. (A) Two-dimensional sheet with a dual-negative (CK7^-^/PAX8^-^) pattern represents mesothelial cells. (B) Three-dimensional large clusters with dual staining for nuclear PAX8 and cytoplasmic CK7. (C) Three-dimensional large clusters with staining for cytoplasmic CK7, but no nuclear PAX8 staining. (D) Two-dimensional sheet with staining for CK7 indicates that the cells are not mesothelial cells.

Each parameter showed significant statistics to the specific primary origin. In terms of three-dimensional (3D) cluster, three tier categories were defined: ‘no 3D cluster’ was defined as no discernable 3D cluster with diffusely dispersed smear; ‘small 3D cluster’ was defined as 3D clusters composed of less than 10 cells at high magnification; and ‘large 3D cluster’ was defined as 3D clusters composed of more than 10 cells at high magnification. Different dual staining was easily recognized in scattered cells, but could be recognized in cell clusters as well (Fig 3). Because background mesothelial cells are completely negative for the dual markers, any stained cells raise suspicion of malignancy.

### Three-dimensional cluster and immunocytochemistry patterns based on the primary site

**Fig 4:**
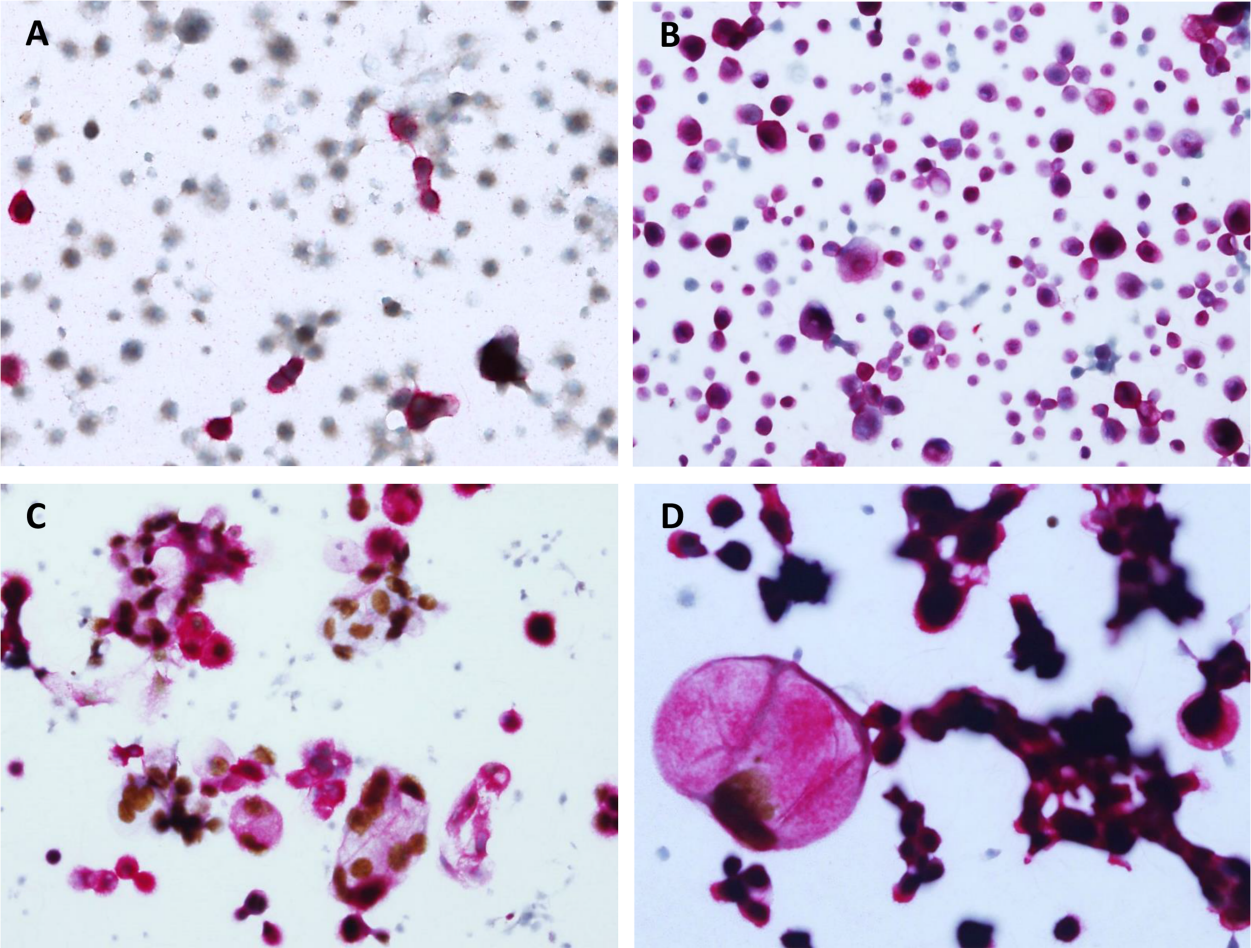
Typical immunocytochemistry patterns based on primary origins. Three parameters, three-dimensional (3D) clusters and dual staining for CK7 and PAX8, can identify the primary origin. Each panel represents different patterns of dual staining. (A) Stomach cancer: most cells are scattered and show CK7^+^/PAX8^-^ immunoprofile. (B) Gastrointestinal tract, including stomach and pancreatobiliary tract cancer: mostly small 3D cluster with scattered cells are CK7^+^/PAX8^-^. (C and D) Female genital tract, including ovarian cancer: large 3D cluster with scattered cells are CK7^+^/PAX8^+^. There is positive staining for nuclear PAX8-DAB in the cell with the large cytoplasm.

In malignant ascites from stomach cancer, 3D cluster showed an inverse correlation to probability of stomach origin. However, it was not significant (OR = 0.052 relative to no cluster; *P* = 0.239) in univariate analysis. However, stomach cancer showed a tendency toward to be associated with no 3D cluster in multivariate analysis (OR = 0.144; *P* = 0.097). When combined with dual ICC, 3D cluster pattern became a significant factor associated with the primary origin (Fig 4A). Small 3D clusters were observed in some of stomach cancer and the majority of pancreatobiliary tract cancers. In dual ICC, these cases showed CK7 expression with PAX8 negativity (Fig 4B).

Large 3D cluster showed a significant correlation with ovarian cancer as the primary origin (OR = 11.16; *P* < 0.0001) (Fig 4C and 4D). Besides ovarian cancer, colon and lung cancers showed large 3D clusters dominant malignant ascites. However, ICC pattern of these two cancers differed and only lung cancer showed a significant correlation (OR = 16.67; *P* = 0.0069). The morphological and ICC features of malignant peritoneal fluid samples are summarized in Table 2.

**Table 2:** The summary of morphological and immunocytochemistry patterns.

Primary site	3D Cluster	Cluster Type	CK7	PAX8	Statistical Significance for Combined Result of Cluster Type and ICC Result
Stomach	None (single cells)	**-**	+	-	AUC = 0.8699
Ovary	Yes	Large	+	+	AUC = 0.9812
Pancreatobiliary	Yes	Small	+	-	AUC = 0.8772
Breast/Lung/Colon	Yes	Large	+	-	AUC = 0.882*

* AUC is calculated for cases of which originated from lung.

### Quantification of dual-stained cells in peritoneal fluid

**Fig 5:**
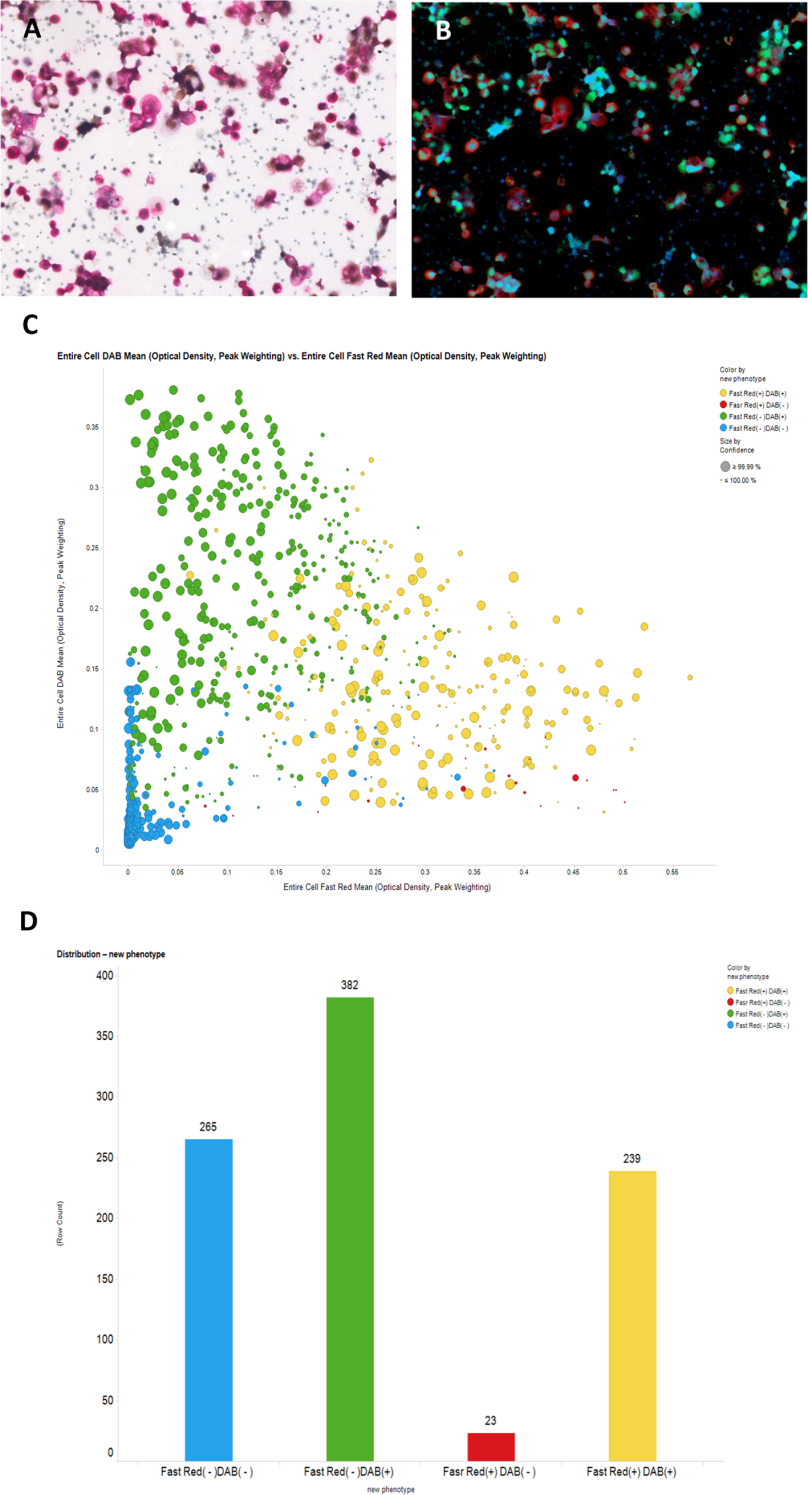
Automatic digitalized interpretation of dual immunocytochemistry. In peritoneal aspirates from patients with ovarian cancer, the suspended cells were very heterogeneous: a mixed population of mesothelial cells and malignant tumor cells. Except for 265 mesothelial cells with dual negative staining (CK7^-^/PAX8^-^), all cells were compatible with malignant cells. Approximately 30% of the cells had the typical dual staining (CK7^+^/PAX8^+^) pattern.

**Fig 6:**
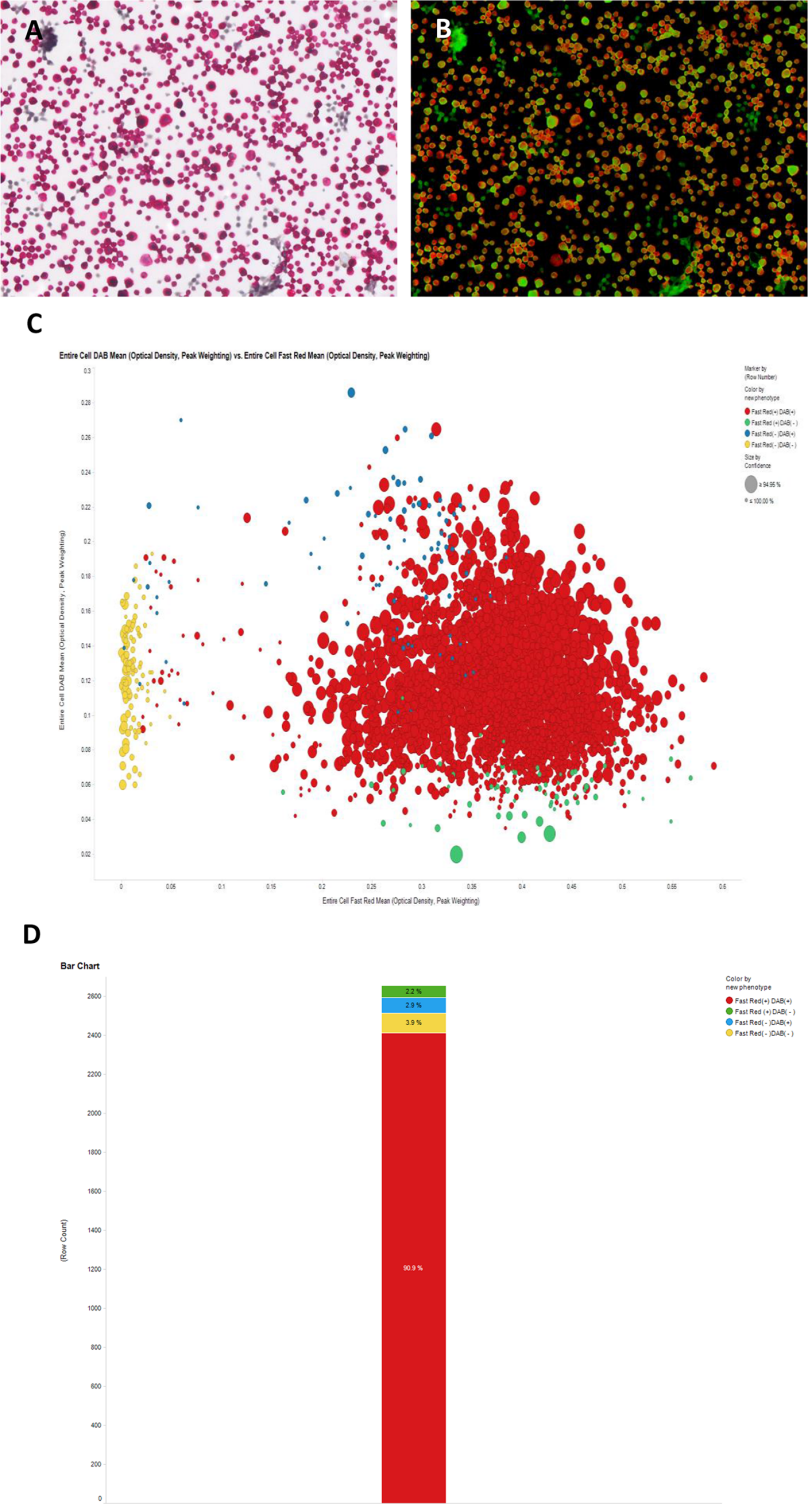
Automatic digitalized interpretation of dual immunocytochemistry. In most cases of ovarian cancer, cells with the typical dual staining (CK7^+^/PAX8^+^) pattern represented over 90% of the cell population.

Quantification of dual-stained cells via image analyzer was performed in malignant peritoneal fluids with ovarian cancer. In ovarian cancer patients, quantification of dual-stained cells varies from less than 30% to greater than 90% of the cell population (Figs 5 and 6). Dual-positive fractions are variable because the cells suspended in ascites are heterogeneously mixed and population ratios can differ between patients. Generally, the value is not accepted for quantification and is discarded in cases of dual-positive staining of less than 5% of the total cell population.

### Receiver operating characteristic curve analysis

**Fig 7:**
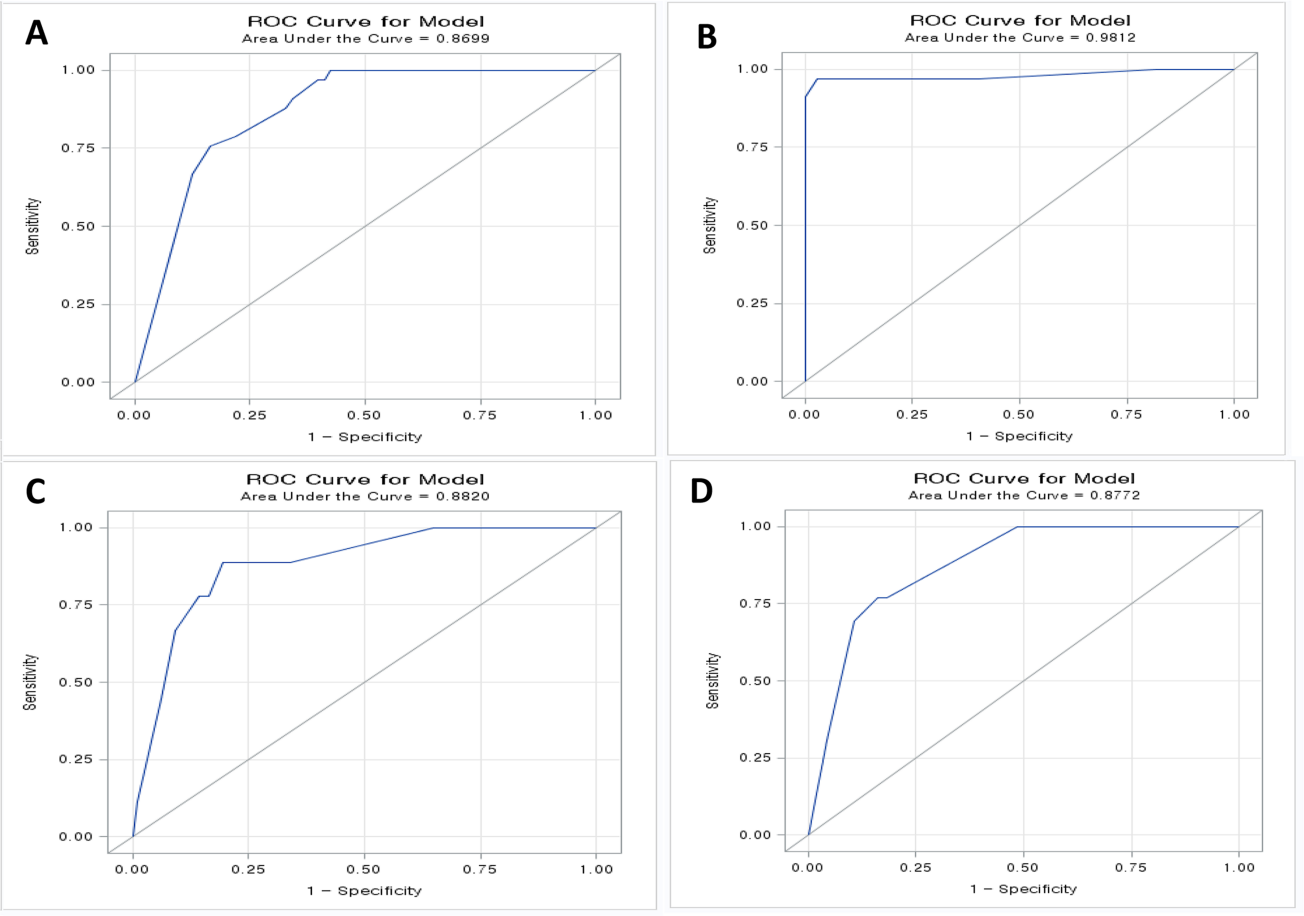
Estimated ROC curve analysis using the three parameters to predict primary origin. (A) In stomach cancer, AUC was 0.8699. (B) In ovarian cancer, the AUC was 0.9812. (C) In lung cancer, the AUC was 0.882. (D) In pancreatobiliary tract cancer, the AUC was 0.8772.

Multivariate analysis of factors associated with 33 stomach cancer patients revealed that small cluster was significantly lower (OR = 0.144, *P* = 0.0097) than no cluster. When combined with the three variables, AUC composed of no 3D cluster CK7^+^, and PAX8^-^ was 0.8699 in ROC curve analysis (Fig 7A). In multivariate analysis for 34 ovarian cancer patients, positive indications of all three parameters were significant for predicting ovarian cancer as the primary site. PAX8 expression was absolutely required for predicting ovarian cancer (OR ≥ 999.99, *P* < 0.001). The AUC was 0.9812 in ROC curve analysis (Fig 7B). Lung cancer samples showed overlapping patterns of three parameters. However, large 3D cluster, CK7^+^, and PAX8^-^ showed a relatively high AUC of 0.882 (Fig 7C). Although colon and breast cancer samples had no AUCs over 0.8 in any modality, the breast cancer samples had overlapping data with lung cancer. In multivariate analysis for 13 pancreatobiliary tract cancer patients, AUC composed of small 3D clusters, CK7^+^, and PAX8^-^ was 0.8772 in ROC curve analysis (Fig 7D).

### Proposal of diagnostic decision tree

Classification and Regression Tree (CART) analysis was performed to predict the possibility of primary site of peritoneal fluid samples. No 3D cluster and CK7^+^ could sufficiently predict the possibility of stomach cancer as the primary tumor. PAX8 expression in peritoneal fluid samples was necessary for the prediction of ovarian cancer.

**Fig 8:**
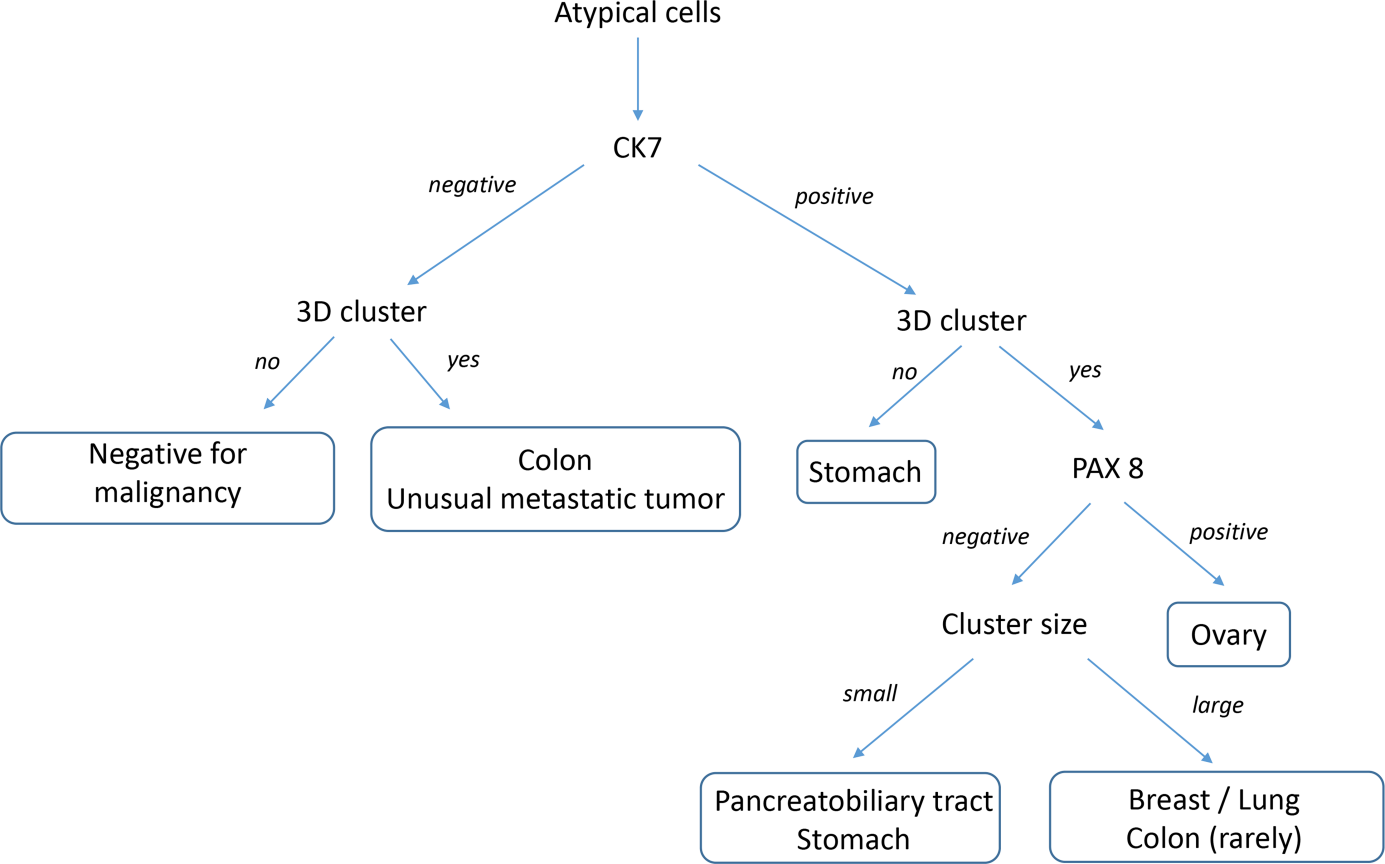
Diagnostic decision tree for peritoneal fluid cytology utilizing cellular cluster and immunocytochemistry patterns to identify primary cancers which have metastatized to peritoneal fluids.

From these results, the combination of 3D cluster status and ICC with CK7 and PAX8 permitted the ability to construct a diagnostic decision tree (Fig 8) to help identify the primary cancer which metastasized into the peritoneal cavity. It permits a simple and strightfoward method to help identify the primary tumor in these peritoneal fluid cytology specimens.

## Discussion

LBC is better for proper interpretation than conventional cytology due to increased cellular yields and clearer backgrounds.[1] When the third space effusion is applied to LBC, these merits are accentuated and read times are decreased. Moreover, the greatest advantage of LBC is its easy application for molecular ancillary studies, including ICC. However, there is no strong supporting evidence for widely accepted biomarker panels for LBC in effusion fluids.[12]

In addition to determining whether cells within the effusion are benign or malignant, additional information as to the suspected origin based on evidence from immunopanels or molecular panels will aid in the management and treatment of patients with unknown primary dissemination. Currently, many studies have sought to determine the best marker for differentiating between mesothelial cells and adenocarcinoma over the broad spectrum.[2] ICC using LBC has been widely used in vaginal cytology with Ki-67 and p16,[4,5] and later adopted in urine cytology for aiding in identification of high-grade urothelial carcinoma.[6] However, no supportive data on dual staining using other markers has been documented. Furthermore, customized dual staining is difficult to optimize, which makes LBC less popular.

In the current study, we examined malignant ascites via peritoneal cytology. The five most common primary sites within our cohort were 1) stomach, 2) ovary, 3) pancreatobiliary tract, 4) breast, and 5) lung cancers. We were able to differentiate primary site of peritoneal fluid samples based on the combination of dual ICC using CK7 and PAX8, and 3D cluster patterns. However, hurdles for dual ICC need to be overcome in both the technical and interpretational aspects.[13] Specifically, the first applied color becomes weak or even completely lost following application of the second color. Therefore, we optimized the technical aspect of dual staining by first performing nuclear staining (PAX8 with DAB) and then performing cytoplasmic or membranous staining (CK7 with Fast Red).

The expression of CK7, when combined with CK20 expression, can be used to determine primary origin.[14] A previous study showed that for CK7^+^ tumors, the staining distribution of CK7 was frequently diffuse (in > 50% of positive tumor cells) in primary ovarian, upper gastrointestinal tract (including pancreatobiliary tract), and endocervical tumors, whereas the CK7 staining distribution was often focal (in < 50% of positive tumor cells) when present in colorectal and appendix carcinomas.[15]

Our collection contained only 4 cases of cancer of the lower gastrointestinal tract. Although CK7 expression in colon cancer is rare,[15,16] all of 4 cases showed relatively lower intensities of CK7 staining. Thus, in our study, large 3D cluster and weak CK7 expression in SurePath^TM^ analysis raised the possibility of colorectal origin in malignant ascites. Although CK7 is a very sensitive marker for screening malignant cells in third cavity fluids, which is essential for the first panel, it can be expressed at variable levels in a broad-spectrum of cancers, including pancreatic, biliary tract, colon, breast, and lung, to evoke malignant ascites.

PAX8 is a nuclear marker which is commonly expressed in epithelial tumors of the thyroid and parathyroid glands, kidney, thymus, and ovary.[10] In terms of malignant peritoneal cytology, the presence of cells with ovary and kidney origins is possible. In our cohort, we collected peritoneal cytology. However, PAX8 staining was non-existent in the case of metastatic carcinoma from the kidney and could be ignored. Henceforth, PAX8 staining was very specific to ovarian origin in malignant ascites, and can be considered the best choice for combining with cytoplasmic staining.

The 3D clusters that were tightly composed of atypical cells appeared strikingly upward and forward compared to the diffusely dispersed mesothelial cells in the background of the SurePath^TM^ technique. This is a unique merit not available with conventional cytopreparatory smears or even with other LBC techniques. In the present study, cluster alone was not sufficient for determination of primary origin. However, in some specific primary origins, cluster pattern aided in the determination of the primary origin. Cells from ovarian and lung cancers tend to form large clusters. Cells from pancreatic and biliary tract cancers tend to form small aggregates, whereas stomach cancer seldom forms aggregates.

In ROC curve analysis to determine diagnostic biomarkers for the prediction of primary site in malignant ascites, diagnostic algorithm using these three parameters showed high AUCs over 0.8. The power to predict the primary site was best in ovarian cancer (AUC of 0.9812), which was specific to large 3D cluster/CK7^+^/PAX8^+^ for differentiating ovarian cancer from gastrointestinal tract cancers. It is also plausible to differentiate stomach cancer from pancreatobiliary tract cancers, because both revealed quite different panels that seldom overlapped.

Screening based on a blind eye is generally possible, but sometimes can be misleading resulting in false negative or false positive diagnoses. The average difficulty of interpretation in dual ICC was reported as 1.06, compared to 2.95 with the one-color method, suggesting that the one-color method is approximately three times as difficult to interpret.[12] The difficulty index increases in cases with few cells and cases with a predominance of isolated tumor cells. For reproducible and accurate interpretations, automatic image analyzing tools are adopted.[7] Cases with double-negative staining do not require an automatic analyzer, while other cases require digitalized fractionation of each cell type. Using the image analyzer, most of the ovarian and stomach cancers were clearly shown to be mainly dual positive and dual negative, respectively. However, other types of cancers, such as pancreatic, lung, and breast, showed wide ranges of dual staining. With principle of omission of values less than 5%, major fractions were accepted as true signals.

The current reporting system is merely negative or positive for malignancy. However, this upgraded technique can provide the following additional outcome of “*Positive for malignancy likely originating from the stomach (or ovary or pancreatobiliary tract)*. *Clinical survey for stomach (or ovary or pancreatobiliary tract) evaluation is suggested*.”. Therefore, we suggest the diagnostic algorithm as follows: 1) When no or only small 3D clusters are visible in LBC with only CK7 positivity, we recommend “*Positive for malignancy likely originating from the stomach*. *Clinical survey for stomach evaluation is suggested*.”. 2) When many large 3D clusters are visible in LBC with CK7 and PAX8 positivity, we recommend “*Positive for malignancy likely originating from the uterus or ovary*. *Clinical survey for gynecologic evaluation is suggested*.”. 3) When many small 3D clusters are visible in LBC with only CK7 positivity, we recommend “*Positive for malignancy likely originating from the pancreatobiliary tract*. *Clinical survey is suggested*.”. 4) When many large 3D clusters are visible in LBC with only CK7 positivity, we recommend “*Positive for malignancy likely originating from breast*, *lung*, *or colon*. *Clinical survey is suggested*.”. When additional details as to the primary origin are requested, a second panel including TTF-1, CDX-2, and mammoglobin can be performed to further narrow the broad category.

## Conclusions

LBC (CytoRich using the SurePath^TM^ method) involving dual ICC with CK7 and PAX8 staining and 3D cluster patterns as a surrogate biomarker can provide information as to the primary origin in patients with malignant ascites of unknown primary origin.

## Supporting information

S1 Table Primary site, cytomorphological features and the results of dual immunocytochemistry of 106 cases. (XLSX)
